# Evaluation of Sterilisation Techniques for Regenerative Medicine Scaffolds Fabricated with Polyurethane Nonbiodegradable and Bioabsorbable Nanocomposite Materials

**DOI:** 10.1155/2018/6565783

**Published:** 2018-10-03

**Authors:** Michelle Griffin, Naghmeh Naderi, Deepak M. Kalaskar, Edward Malins, Remzi Becer, Catherine A. Thornton, Iain S. Whitaker, Ash Mosahebi, Peter E. M. Butler, Alexander M. Seifalian

**Affiliations:** ^1^UCL Centre for Nanotechnology & Regenerative Medicine, University College London, Royal Free London NHS Foundation Trust, Pond Street, London NW3 2QG, UK; ^2^The Charles Wolfson Center for Reconstructive Surgery, Royal Free London NHS Foundation Trust Hospital, London, UK; ^3^Department of Plastic Surgery, Royal Free London NHS Foundation Trust, Pond Street, London NW3 2QG, UK; ^4^Reconstructive Surgery & Regenerative Medicine Group, Institute of Life Science, Swansea University Medical School, Singleton Park, Swansea SA2 8PP, UK; ^5^Welsh Centre for Burns & Plastic Surgery, ABMU Health Board, Heol Maes Egwlys, Swansea SA6 6NL, UK; ^6^Polymer Chemistry Laboratory, School of Engineering and Materials Science, Queen Mary University of London, Mile End Road, London E1 4NS, UK; ^7^Director/Professor Nanotechnology & Regenerative Medicine, NanoRegMed Ltd., The London BioScience Innovation Centre, London NW1 0NH, UK

## Abstract

An effective sterilisation technique that maintains structure integrity, mechanical properties, and biocompatibility is essential for the translation of new biomaterials to the clinical setting. We aimed to establish an effective sterilisation technique for a biodegradable (POSS-PCL) and nonbiodegradable (POSS-PCU) nanocomposite scaffold that maintains stem cell biocompatibility. Scaffolds were sterilised using 70% ethanol, ultraviolet radiation, bleach, antibiotic/antimycotic, ethylene oxide, gamma irradiation, argon plasma, or autoclaving. Samples were immersed in tryptone soya broth and thioglycollate medium and inspected for signs of microbial growth. Scaffold surface and mechanical and molecular weight properties were investigated. AlamarBlue viability assay of adipose derived stem cells (ADSC) seeded on scaffolds was performed to investigate metabolic activity. Confocal imaging of rhodamine phalloidin and DAPI stained ADSCs was performed to evaluate morphology. Ethylene oxide, gamma irradiation, argon plasma, autoclaving, 70% ethanol, and bleach were effective in sterilising the scaffolds. Autoclaving, gamma irradiation, and ethylene oxide led to a significant change in the molecular weight distribution of POSS-PCL and gamma irradiation and ethylene oxide to that of POSS-PCU (p<0.05). UV, ethanol, gamma irradiation, and ethylene oxide caused significant changes in the mechanical properties of POSS-PCL (p<0.05). Argon was associated with significantly higher surface wettability and ADSC metabolic activity (p<0.05). In this study, argon plasma was an effective sterilisation technique for both nonbiodegradable and biodegradable nanocomposite scaffolds. Argon plasma should be further investigated as a potential sterilisation technique for medical devices.

## 1. Introduction

Synthetic biomaterials are being used to replace the extracellular matrix to restore damaged and failing tissues and organs [1]. Amongst biomaterials, polymeric scaffolds have gained significant popularity due to their ease of fabrication and versatility [1]. Polymeric scaffolds for tissue engineering are either manufactured aseptically or sterilised after processing [2, 3]. For economical and practical reasons, the latter strategy has been employed with polymeric scaffolds intended for in vivo use and is considered a more realistic approach to achieve sterile implantable scaffolds [1, 2]. Nevertheless, the challenge remains to determine an efficient and nondestructive sterilisation procedure for polymer scaffolds that preserves their structure and surface properties [3]. Sterilisation techniques may influence a material's structural, chemical, and biological properties; thus it is important to ensure the modality implemented does not affect biocompatibility [3]. Sterilisation of biomaterials accepted by the FDA for medical devices includes ethylene oxide, autoclaving, and gamma sterilisation [4]. The success of an implant for sterilisation is dependent not only on the implant remaining sterile, but also on achieving sterility without adversely affecting the material's properties. Different sterilisation agents have shown that they can attack polymers causing hydrolysis, melting, or depolymerisation [5, 6].

Our group have developed and patented two families of nanocomposite polymers for the development of organs and tissues [7–9]. The nonbiodegradable polymer incorporates POSS nanoparticles into polycarbonate-based urea-urethane (POSS-PCU, UCL-Nano). Its biodegradable counterpart modifies poly(caprolactone urea-urethane) POSS-PCL. Understanding an appropriate sterilisation technique for POSS-PCU and POSS-PCL is crucial for translation to clinical practice. A brief previous study compared three techniques of sterilising POSS-PCU and POSS-PCL scaffolds including autoclaving, gamma irradiation, and ethanol [7]. Autoclaving was found to be effective in maintaining sterilisation of the scaffolds without degrading the material. The first authors of this paper have also demonstrated that bleach may be useful for sterilising POSS-PCL scaffolds compared to ethanol and autoclaving sterilisation [8]. Adipose derived stem cells (ADSCs) were shown to adhere to the POSS-PCL scaffolds following ethanol and bleach sterilisation [8]. Lastly, a study comparing the effects of autoclave, microwave, antibiotics, and 70% ethanol sterilisation on POSS-PCL scaffolds found ethanol to be a suitable sterilisation technique with maintained fibroblast attachment [9]. The aim of this study was to compare all available sterilisation techniques for POSS-PCU and POSS-PCL in a single study including a new method using argon plasma sterilisation, building on previous studies, to understand the optimal sterilisation technique for nanocomposite scaffolds.

An increasingly popular method of modifying the surface functionality to enhance cell behaviour on scaffolds is plasma modification [10, 11]. Plasma consists of electrons, ions, energy rich neutrals, molecules, fragments, atoms, and photons. It can be under low, atmospheric, or high pressure. The distinct behaviour of gases led to the suggestion that plasma is the “fourth state of matter” [12]. Plasma modification (PM) is an easy, reliable, clean way of creating reactive functional groups on the surfaces of biomaterials and for creating anchoring sites for further chemical reactions [12].

This study compared ethylene oxide, argon plasma, bleach, antibiotic/antimycotic, ethanol, ultraviolet radiation, autoclaving, and gamma irradiation sterilisation procedures for morphological alteration, chemical damage, effects on polymer degradation, and biocompatibility. The viability of ADSCs was assessed following the sterilisation of POSS-PCU and POSS-PCL scaffolds to assess biocompatibility of the sterilisation techniques.

## 2. Materials and Methods

### 2.1. Polymer Synthesis

#### 2.1.1. POSS-PCU

The nanocomposite scaffolds were manufactured as previously described [7, 13]. In brief, polycarbonate polyol, 2000 mwt, and* trans*-cyclohexanechloroydrinisobutyl-silsesquioxane (Hybrid Plastics Inc.) was heated to 135°C and then cooled to 70°C. Flakes of 4,4′-methylenebis (phenyl isocyanate) (MDI) were then added to the mixture, at 75–85°C for 90 minutes to form the prepolymer. Then* N*,*N*-dimethylacetamide (DMAc) was to form a solution. Further chain extension was completed by the drop-wise addition of ethylenediamine and diethylamine in DMAc. This then created the POSS-modified polycarbonate urea-urethane in DMAc. All chemicals and reagents were purchased from Aldrich Limited, Gillingham, UK.

#### 2.1.2. POSS-PCL

POSS-PCL nanocomposites solution was manufactured as described previously [7]. In brief, polycaprolactone diol (2000 g/mol) and* trans*-cyclohexanechlorohydrinisobutyl-polyhedral oligomeric silsesquioxane (POSS) were mixed and heated to 135°C. Then 9.4 g of 4,4′-methylenebis(cyclohexylisocyanate) was added to form a prepolymer. Following this, 100 g of DMAC was added to the prepolymer. Chain extension was performed by drop-wise addition of 1 g of ethylenediamine in 80 g of dry DMAC. Following this, 2 g of 1-butanol in 5 g of DMAC was added to form the nanocomposite. All chemicals and reagents were purchased from Aldrich Limited, Gillingham, UK.

#### 2.1.3. Sample Preparation

Polymers were fabricated as 3D scaffolds using the phase separation/particulate-leaching technique as described previously [13]. Firstly, NaCL (200-250 *μ*m) was dissolved in POSS-PCL and POSS-PCU in DMAc containing Tween-20 surfactant (1:1 weight ratio). The solution was dispersed and degassed in a Thinky AER 250 mixer (Intertonics, Kidlington, UK). The polymer mixture was spread onto steel moulds. The moulds were then washed in deionised water to dissolve the solvent and DMAC for 7 days. Following washing, polymer sheets with 700-800 *μ*m thickness were manufactured. For cell culture analysis, 16 mm polymer disks were cut from the sheets using a steel manual shape cutter.

### 2.2. Sterilisation

#### 2.2.1. Gamma Irradiation

Scaffolds were irradiated with a dose of 25 kGy at room temperature, using a ^60^Co gamma-ray source (Synergy Health, Swindon, UK). Scaffolds were exposed on a continuous path for 10 hours as described previously [7].

#### 2.2.2. Autoclaving

The scaffolds were exposed to steam at 121°C for 15 minutes at pressures of 115 kPa as previously described [7].

#### 2.2.3. Ethanol

Polymer scaffolds were submerged in 70% (v/v) ethanol on a roller mixer for 30 minutes as previously described [8]. Following alcohol sterilisation, scaffolds are washed in sterile deionised water on a roller for 15 minutes, which is then repeated five times.

#### 2.2.4. Plasma

Scaffolds are placed in 24-well plates for treatment by LF (radio frequency) argon plasma generator operating at 40 KHz at 100 W. Scaffolds then enter the chamber of the electrode-less, glow discharge apparatus, which is purged 3 times with argon gas (99.99% purity, BOC, UK) for 2 minutes. The chamber is then evacuated to 1.0 Torr. Plasma is then ignited by a radio frequency excitation source and was maintained at 100 W for 5 minutes. The scaffolds were treated with plasma and immediately seeded with cells for* in vitro* analysis to prevent hydrophobic recovery of the scaffolds.

#### 2.2.5. Ethylene Oxide

The ethylene oxide sterilisation is initiated with a preconditioning of the samples which is carried out at 41°C for 13 hours at 42% humidity. The sterilisation step is then performed by 100% ethylene oxide atmosphere at 49°C for 2 1/2 hours. Scaffold are then in air for 9 hours at 43°C.

#### 2.2.6. Ultraviolet Irradiation

Scaffolds were UV irradiated by placement in a UV decontamination device (40 watt wavelength 254 nm, mean density 15 kJ/cm^2^) for 3 hours as previously described [8].

#### 2.2.7. Antibiotic Antimycotic Treatment

Scaffolds were placed in a 1% (v/v) antibiotic antimycotic solution (10 000 U/mL penicillin G, 10 mg/mL streptomycin sulphate, and 25 mg/mL amphotericin B diluted in sterile phosphate buffered saline (PBS)) for 24 hours at 4°C. Following sterilisation, the scaffolds were washed five times with sterile deionised water on a roller for 15 minutes each time.

#### 2.2.8. Bleach (SDIC)

The scaffolds were submersed in 1000 ppm slow chlorine releasing compound sodium dichloroisocyanurate dihydrate (SDIC) at room temperature for 20 minutes as described previously [8]. The scaffolds are then washed in sterile deionised water daily for 7 days. The removal of remaining SDIC was confirmed by pH testing.

### 2.3. Material Characterisation

#### 2.3.1. Tensiometry

Tensiometry to evaluate the mechanical properties of the scaffolds following sterilisation was performed as described previously [13]. In brief, dumbbell-shaped scaffolds (dimensions of 10 × 2 mm) were tensile loaded at a loading speed of 100 mm/min (*n* = 6) using an Instron 5565 (High Wycombe, Bucks, UK). Young's modulus of elasticity at the 0-25% portion of the curve, maximum tensile strength, and elongation at break were calculated. Statistical differences in the tensile properties between sterilisation techniques were evaluated using two-way ANOVA with post hoc Turkey test.

#### 2.3.2. Attenuated Total Reflectance Fourier Transform Infrared Spectroscopy (ATR-FTIR)

FTIR spectrophotometer was used to analyse changes in the surface chemistry of the scaffolds treated with the different sterilisation techniques as previously described [8]. Chemical groups were detected using attenuated total reflectance (ATR)-FTIR mode (Jasco FT/IR 4200 Spectrometer (JASCO Inc., USA)) (n=6). FTIR testing parameters were recorded at 20 scans at a 4 cm-1 resolution with a wavenumber range of 600 cm-1 to 4000 cm-1.

#### 2.3.3. Gel Permeation Chromatography (GPC)

Molecular weight averages and polymer dispersity were determined by GPC as previously described [8] (n=6). In brief, samples were prepared to a 1 mg/mL concentration and passed through a 0.22 *μ*m nylon filter. GPC analysis was conducted on the Agilent 1260 infinity system using 2 PLgel 5 *μ*m mixed-D columns (300 × 7.5 mm), a PLgel 5 mm guard column (50 × 7.5 mm), a differential refractive index (DRI), and variable wavelength detector (VWD). Statistical differences in the GPC analysis between sterilisation techniques were evaluated using two-way ANOVA with post hoc Turkey test.

#### 2.3.4. Water Contact Angle Measurements

The static water contact angle of the scaffolds was performed as described previously [13]. In brief, the water contact angle was analysed using the sessile drop method (DSA 100 instrument (KRUSS, Germany)). A 5 *μ*l volume of deionised water was used in all experiments. Measurement of a single drop was performed on six independent scaffolds (n=6). The average contact angle was calculated using the KRUSS drop shape software (version 1.90.0.14).

#### 2.3.5. Scanning Electron Microscopy (SEM)

The surface of the scaffolds treated with the different sterilisation techniques was analysed using SEM as described previously [8] (n=6). Scaffolds were dehydrated in acetone prior to drying overnight. The scaffolds were then mounted on aluminium pin stubs using sticky carbon tape. After coating the scaffolds with a thin layer of Au/Pd (approximately 2 nm thick) using a Gatan ion beam coater the surface of the scaffolds was imaged with a Carl Zeiss LS15 Evo HD SEM.

### 2.4. Cytotoxicity

#### 2.4.1. ADSC Isolation and Seeding

ADSCs were isolated from adipose tissue according to the method described by Zuk et al. with modifications as previously described [8, 14, 15]. In brief, following removal of fibrous tissue adipose tissue was cut into small pieces of < 3 mm^3^. The tissue was then further digested in Dulbecco's Modified Eagle's Medium/Nutrient Mixture F-12 Ham (DMEM/F12) containing 300 U/mL crude collagenase I (Invitrogen, Life Technologies Ltd., Paisley, UK) for 30 minutes in an incubator (37°C, 5% CO_2_). Following filtration through 70 *μ*m Cell Strainers (BD Biosciences, Oxford, UK) the samples underwent centrifugation (290 × G, 5 min). Then the ADSC-rich cell preparation formed a pellet at the bottom of the tube. The ADSC cells were cultured for up to 2 passages. When the ADSCs reached approximately 80% confluence, subculture was performed through trypsinisation. For cell cultures analysis, 1.5 X 10^4^ ADSCs at passage 2 were seeded on each polymer disk following sterilisation. Written consent was taken from all patient donors in the study and was approved by the North Scotland ethical review board, reference number 10/S0802/20.

#### 2.4.2. AlamarBlue

AlamarBlue, viability assay, was performed as described previously [8, 13]. Briefly following incubation of scaffolds with complete medium for 24 hours, scaffolds were seeded with 1.5 X 10^4^ ADSC per well. At 1, 3, 7, and 14 days medium was removed and 10% AlamarBlue prepared in fresh media was added for 3 hours. Following incubation, AlamarBlue fluorescence was quantified at the respective excitation and emission wavelength of 540 and 595 nm. The mean fluorescent units for the six replicate cultures of three individual experiments were calculated for each exposure treatment and the mean blank value was subtracted from these.

#### 2.4.3. Immunofluorescence: Rhodamine Phalloidin and DAPI

To study adhesion and morphology of the ADSC onto the scaffolds, immunocytochemistry morphology staining was performed as described previously [8, 13]. At 24 hours, the media was removed and the cells were washed with PBS three times. Following this, cells were fixed with 4% (w/v) paraformaldehyde for 15 minutes. The scaffolds were then washed thrice in PBS/0.1% Tween-20 and washed in 0.1% tritonX100 to improve permeability for 5 minutes. The scaffolds were then stained with rhodamine phalloidin dye (Molecular Probes®, Life Technologies, Paisly, UK) in the ratio 1:40 (dissolved in 1 mL of methanol) in PBS for 40 minutes. Following washing the nuclei was stained with DAPI (Molecular Probes®, Life Technologies, Paisley, UK). The ADSCs on the scaffolds were visualised using confocal microscopy Zeiss LSM 710 (Zeiss, Jena, Germany). Image J (National Institute of Health, NIH) software was used to determine circularity of the ADSCs on the scaffolds.

### 2.5. Sterility Testing

All samples were tested for the effectiveness of sterilisation as previously described [8]. In brief, scaffolds were immersed in tryptone soya broth (TSB) and fluid thioglycollate medium (THY) for cultivation of microorganisms (Wickham Laboratories, Hampshire) for 7 days. Sterile broth was considered the negative control and unsterilised samples as the positive control. Both broths were macroscopically observed every 1–3 days for clouding as an indicative of contamination and ineffective sterilisation. A clear broth was considered to have no infection and an effective sterilisation of the samples (*n* = 9).

### 2.6. Statistical Analysis

All statistical analyses were performed using Prism software (GraphPad Inc., La Jolla, USA). Means and standard deviations were calculated from numerical data. In figures, bar graphs represent means, whereas error bars represent 1 standard deviation (SD). A *p* value of ≤ 0.05 was defined as significant. The exact statistical analysis performed for each dataset is described in the figure legend.

## 3. Results

### 3.1. Material Characterisation

#### 3.1.1. Visual Inspection after Sterilisation

All samples withstood treatment with UV, antibiotic/antimycotic, bleach, and plasma treatment. Although the POSS-PCU samples were unaffected by the autoclaving process, the POSS-PCL samples were destroyed; therefore, it was not possible to examine the autoclaved POSS-PCL samples further. Both the POSS-PCU and POSS-PCL samples held up well against gamma irradiation with slight discolouring/yellowing of the POSS-PCU samples being observed. Ethylene oxide gas caused slight yellow discolouring and ethanol visible deformation of POSS-PCL samples.

#### 3.1.2. Tensiometry

Quantitative values of mechanical properties of POSS-PCL and POSS-PCU samples subjected to ethanol, bleach (SDIC), plasma, ethylene oxide gas, UV radiation, antibiotic/antibiotic treatment, gamma irradiation, autoclaving (only POSS-PCU), and unsterilised controls for each sterilisation method are presented in Table 1. No significant difference in elongation at break, Young's modulus, or maximum stress was seen between any of the POSS-PCU samples. The ultimate tensile stress for POSS-PCL increased from 0.56±0.08 MPa in control samples to 1.71±0.19 MPa after ethylene oxide gas treatment, to 1.45±0.04 after UV radiation, and to 1.17±0.15 after gamma irradiation (p < 0.05). This increase in tensile stress of ethylene oxide treated POSS-PCL translated itself to Young's modulus, which was also significantly increased compared to control (0.40±0.11 versus 0.18±0.0, p < 0.05). Compared to control POSS-PCL, UV radiated POSS-PCL and ethanol had a significantly shorter elongation at break (p < 0.05).

#### 3.1.3. Water Contact Angle Measurements

**Figure 1:**
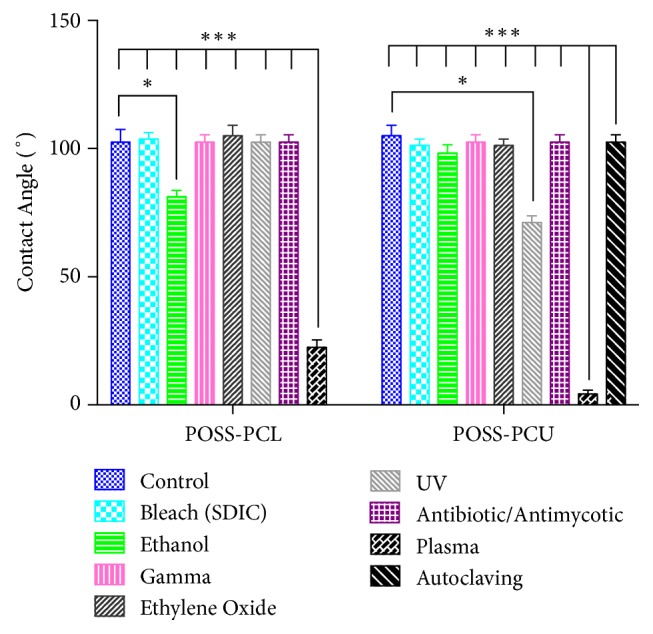
**Contact angles of POSS-PCL and POSS-PCU samples measured after different sterilisation techniques.** One-way ANOVA and Turkey's multiple comparison test was used to show statistical significance. *∗* indicates statistically significant differences (*∗*p < 0.05 and ∗∗∗p < 0.001).

The difference in surface hydrophilicity of POSS-PCL and POSS-PCU samples after each sterilisation technique was assessed by measuring the water contact angle (Figure 1). Plasma treated POSS-PCL and POSS-PCU samples had statistically significant lower contact angles when compared to untreated controls and other sterilisation methods (p < 0.001). Ethanol treatment of POSS-PCL was associated with a significant decrease in contact angles when compared to controls (p < 0.05). Amongst the treated POSS-PCU samples, UV radiation was associated with a statistically significant decrease in contact angle measurements compared to controls (p < 0.05).

#### 3.1.4. Attenuated Total Reflectance Fourier Transform Infrared Spectroscopy (ATR-FTIR)

**Figure 2:**
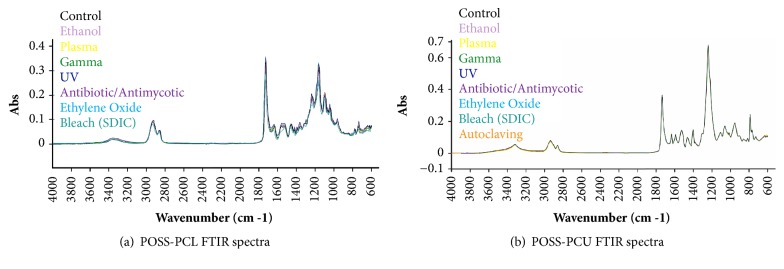
**POSS-PCL and POSS-PCU FTIR spectra after different sterilisation techniques. [a]** SDIC treatment led to a slight decrease in the peaks at 1634 cm^−1^ and 1557 cm^−1^ and an increase in the peak at 1520 cm^−1^ compared to untreated control POSS-PCL.** [b]** No major changes were noted in the FTIR spectra of POSS-PCU samples after different sterilisation methods.

Figure 2 shows the peak assignment in the FTIR spectra of unsterilised control POSS-PCL and POSS-PCU and POSS-PCL and POSS-PCU samples after different sterilisation methods. Bleach (SDIC) treatment led to a slight decrease in the peaks at 1634 cm^−1^ and 1557 cm^−1^ and an increase in the peak at 1520 cm^−1^. POSS-PCL FTIR spectra were not significantly affected by the other sterilisation techniques. FTIR spectra of POSS-PCU samples were unaffected by different sterilisation techniques.

#### 3.1.5. Gel Permeation Chromatography (GPC)

Gel permeation chromatography (GPC) results are summarised in Table 2. The untreated POSS-PCU was found to have a weight average molecular weight (*M*_w_) of 91200 g/mol and a number average molecular weight (*M*_n_) of 47700 g/mol, whereas POSS-PCL had an *M*_w_ of 361100 and *M*_n_ of 141000 g/mol. After ethanol, bleach, UV radiation, and antibiotic treatments there was a negligible impact on *M*_w_, for any of the samples. However, solely amongst POSS-PCL samples, ethanol caused a decrease of 21% in *M*_*n*_, whilst UV increased *M*_*n*_ by 10% in comparison to unsterilised POSS-PCL. No major changes in molecular weight distributions were detected in autoclaved samples of POSS-PCU. However, autoclaved POSS-PCL samples showed a 52% decrease in *M*_w_ and 38% decrease in *M*_n_. Exposure to gamma irradiation had a significant impact on all of the samples. *M*_n_ of POSS-PCU decreased significantly by 16%, whereas *M*_*w*_ increased by 48%. Meanwhile, gamma irradiation decreased both *M*_n_ and *M*_w_ of POSS-PCL by 68% and 58%, respectively. Ethylene oxide increased *M*_w_ of POSS-PCU by 23% and decreased *M*_n_ of POSS-PCU by 28%. Concurrently, it decreased *M*_w_ and *M*_n_ of POSS-PCL by 23% and 31%, respectively. Plasma had no significant impact on the molecular weight distribution of POSS-PCU or POSS-PCL polymers.

#### 3.1.6. Scanning Electron Microscopy (SEM)

**Figure 3:**
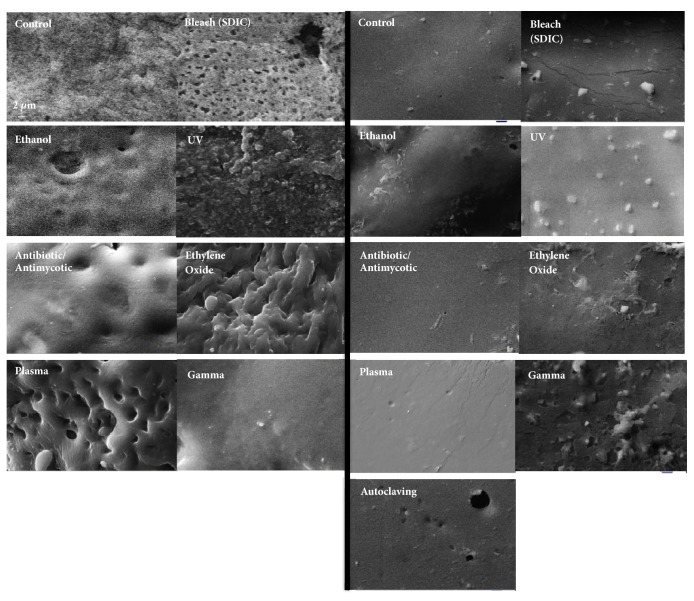
Scanning electron microscopy (SEM) images of POSS-PCL (left) and POSS-PCU (right) surfaces after different sterilisation techniques.

SEM images of POSS-PCL and POSS-PCU after different sterilisation techniques are shown in Figure 3. Unsterilised POSS-PCL sample surface exhibits tufts and pits on the surface. Such tufts were lost after using ethanol, antibiotic/antimycotic, and gamma sterilisation with polymer melting and irregular reformation into larger and flatter ridges. SDIC treatment was associated with a marked increase in pits, whereas UV irradiation was associated with a pronounced increase in the number of tufts. Ethylene oxide was associated with larger tufts, whereas plasma treatment caused larger pits and tufts on the POSS-PCL surface (Figure 3). POSS-PCU samples, generally, showed little surface alterations after sterilisation. Ethanol, SDIC, UV, EO, and gamma were associated with increased tufts. The antibiotic/antibiotic, autoclaving, and plasma sterilisation caused minimal changes on the POSS-PCU surface.

### 3.2. Cell Biocompatibility

#### 3.2.1. AlamarBlue

**Figure 4:**
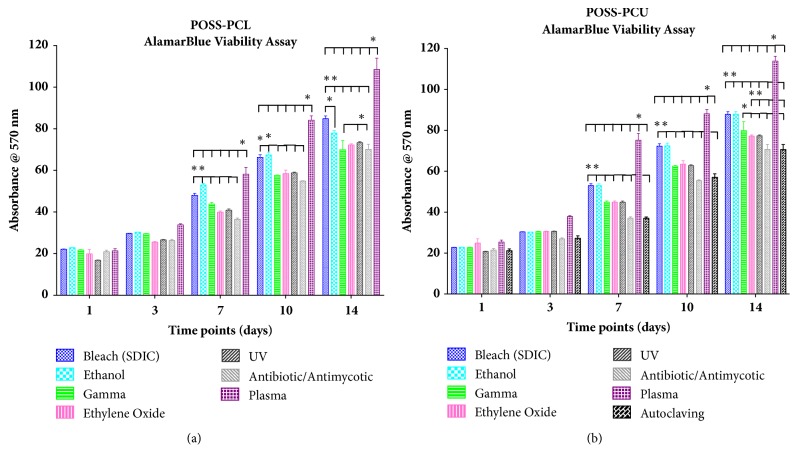
**AlamarBlue viability assay of adipose derived stem cells (ADSCs) after 1, 3, 7, 10, and 14 days of incubation on (a) POSS-PCL and (b) POSS-PCU samples**. Statistical significance was shown using two-way ANOVA and Turkey's multiple comparisons test. *∗* indicates statistically significant differences (*∗*p < 0.05).

The results of the alamarBlue viability assay after 1, 3, 7, 10, and 14 days of incubation are presented in Figure 4. ADSC cultured on POSS-PCU and POSS-PCL samples showed similar metabolic activity. At Days 7 and 10, plasma treated POSS-PCL was associated with the highest ADSC metabolic activity compared to any other sterilisation technique (p < 0.05). Ethanol and bleach (SDIC) sterilised POSS-PCL had a statistically significant higher ADSC metabolic activity compared to ADSC on gamma, ethylene oxide, UV radiation, antibiotic/antimycotic, and autoclaving sterilised POSS-PCL. At Day 14, the same observations were made. In addition, UV radiation sterilised POSS-PCL had a statistically significant higher ADSC metabolic activity compared to ADSC on antibiotic/antibiotic treated and gamma irradiated POSS-PCL (p < 0.05). Bleach (SDIC) sterilised POSS-PCL had a statistically significantly higher ADSC metabolic activity compared to ADSC on ethanol sterilised samples (p < 0.05). Amongst POSS-PCU samples, similar observations were made. At Days 7 and 10, plasma treated POSS-PCU was associated with the highest ADSC metabolic activity compared to any other sterilisation technique (p < 0.05). Ethanol and bleach sterilised POSS-PCU had a statistically significant higher ADSC metabolic activity compared to ADSC on gamma, ethylene oxide, UV radiation, antibiotic/antimycotic, and autoclaving sterilised POSS-PCU (p < 0.05). In addition, gamma, ethylene oxide, and UV radiation treated samples had a statistically significant higher ADSC metabolic activity compared to antibiotic/antimycotic and autoclaving (p < 0.05). At Day 14, the same observations were made. In addition, gamma irradiation was associated with significantly higher ADSC metabolic activity compared to UV and ethylene oxide (p < 0.05).

#### 3.2.2. Immunofluorescence: Rhodamine Phalloidin and DAPI

**Figure 5:**
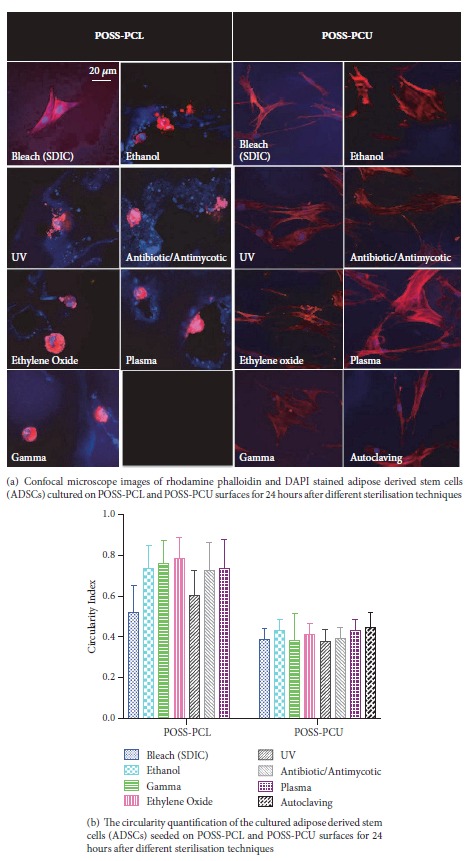


Confocal laser scanning microscopy images indicated that ADSC developed different morphologies when grown on differently sterilised surfaces (Figure 5). Cells grown on bleach sterilised POSS-PCL exhibited a more spread-out phenotype compared to cells grown on surfaces exposed to other sterilisation techniques which demonstrated a more round character. In general, ADSC on POSS-PCL had a more round morphology compared to ADSC on POSS-PCU surfaces. Circularity measurements with Image J software showed that ADSC grown on bleach sterilised POSS-PCL had a significantly less circular morphology compared to ADSC on ethanol, ethylene oxide, gamma, antibiotic/antibiotic, and plasma (p < 0.05) but not on UV sterilised POSS-PCL (Figure 5(a)). There was no statistically significant difference between the morphology of the ADSCs on the POSS-PCU samples (Figure 5(b)).

### 3.3. Sterility Testing

#### 3.3.1. Visual Inspection

The polymeric materials were incubated in TSB and THY for 7 days to test the efficiency of sterilisation with the resultant level of bacterial growth reported in Table 3. THY is a viscous growth medium with reduced oxygen levels, which tests the growth of anaerobic bacteria and other organisms capable of growing in reduced oxygen tension. No evidence of bacterial growth was observed on any of the materials tested after incubation in THY. TSB, however, is a general growth media for aerobic microorganisms and is designed for the growth of aerobic bacteria and yeasts and moulds. Amidst POSS-PCU sterilised samples, there was no sign of infection. Only the unsterilised control samples showed signs of infection. Amongst POSS-PCL samples, all unsterilised control samples and one of nine samples sterilised using UV radiation and antibiotic/antimycotic showed signs of infection. Although there was minimal evidence of bacterial growth in the sterility studies of scaffolds with UV and antibiotic/antimycotic treatment, no evidence was seen in the viability testing or the morphology assessment to invalidate the assays.

## 4. Discussion

Polymer degradation after sterilisation techniques can be assessed using (i) macroscopic characterisation methods, providing information on “bulk” properties such as mechanical performance, (ii) microscopic characterisation, looking at molecular weight and its dispersity, (iii) characterisation of the molecular structure and composition, such as FTIR analysis, and (iv) surface characterisation, i.e., scanning electron microscopy and surface wettability [16]. According to these criteria, in this study, we performed a thorough investigation of the bulk, surface, and molecular properties of POSS-PCL and POSS-PCU scaffolds after several sterilisation techniques, including plasma, gamma irradiation, ethylene oxide, UV radiation, antibiotic/antimycotic, 70% ethanol, and bleach treatments. As the biodegradable counterpart of POSS-PCU, POSS-PCL was considerably more susceptive to changes in the molecular weight distribution, mechanical properties, and surface morphology and chemistry after several sterilisation techniques.

The three leading sterilisation methodologies with FDA approved for medical devices are gamma irradiation, autoclaving, and ethylene oxide. Autoclaving has been the earliest method for the sterilisation of biomaterials. Sterilisation with moist heat in an autoclave is usually performed at temperatures equal to or higher than 121°C; dry heat sterilisation requires considerably higher temperatures to effectively inactivate bacterial spores. The suitability of steam sterilisation has been questioned for polyurethanes, as the high temperature may soften the polymer and deform the material [17]. In this study POSS-PCU material's properties were unaffected as shown by mechanical properties, surface chemistry as shown by contact angle and FTIR, and surface topography as shown by SEM. Polymer degradation was determined by measuring changes in molecular weight and mass immediately after the sterilisation process using GPC analysis. Polymeric biomaterials of low molecular weight present less chemical and mechanical resistance in relation to the same material of higher molecular weight [18]. Autoclaving was an optimal sterilisation technique for POSS-PCU, with no evidence of changes in molecular number and weight. However, POSS-PCL was unsuited to autoclaving as shown by visual changes after sterilisation and changes in molecular weight and number, demonstrating the polymer underwent a degree of hydrolysis. This finding is in accordance with the literature, where biodegradable polymers break down due to the high temperatures of autoclaving [19].

Gamma sterilisation involves ionising radiation from either cobalt 60 isotope or accelerated electrons. Gamma irradiation can generate free radicals in the polymer, causing surface oxidation and subsequent degradation due to chain scission and cross-linking with increasing dosages of radiation [20]. However, gamma irradiation has definite advantages in that it is penetrating and free of residues. Also, material temperatures are only moderately elevated during sterilisation, which is an advantageous feature for the sterilisation of bioresorbable implants, where temperature and dose conditions need close consideration. Microbiological validation experiments according to ISO 11137 [21] have shown gamma irradiation in dry ice at doses of 16 kGy or more effectively inactivates microorganisms. In this study, gamma irradiation was associated with effective sterilisation of POSS-PCL and POSS-PCU scaffolds. Polyurethanes have also shown some degradation after gamma radiation. POSS-PCL was found to have a significant decrease in the molecular weight and number, indicating degradation may have occurred. Several biodegradable polymers have shown to be susceptible to gamma irradiation [19]. Holy et al. found that polylactide-co-glycolide scaffolds had a 50% loss in their molecular weight after gamma irradiation [22].

Gamma irradiation has been shown to cause the release of free radicals in polymers, causing surface oxidation and subsequent degradation of the polymer. Surface oxidation of the polyurethanes is indicated by a strong yellowing of samples, which was also found in this study with some POSS-PCL scaffolds. Despite the degradation and potential surface oxidation of gamma on PCL scaffolds, the water contact of POSS-PCL did not change after gamma sterilisation compared to control. There are reports in the literature that show no change in water contact angle of biomaterials following gamma sterilisation [23, 24]. This could be due to the penetrating action of the gamma sterilisation affecting bulk properties rather than surface properties [23, 24].

Gamma irradiation showed no changes in material properties including mechanical, surface chemistry, or molecular number for POSS-PCU scaffolds. However, SEM did show some surface changes after gamma sterilisation on POSS-PCU. Although suitable for POSS-PCU, POSS-PCL was unsuitable for gamma sterilisation with significant changes in molecular weight and number.

EtO is the final sterilisation technique, which has been approved for medical implants. EtO is a liquid below 11° so in contrast to steam sterilisation it is a low temperature method [17]. In addition to being toxic and explosive causing a significant occupational health and safety hazard, there are concerns on removing all traces of the EtO from the implant after sterilisation [15]. In this study, SEM showed some surface material changes after EtO without changing the bulk properties including tensile strength or molecular weight for POSS-PCU. For POSS-PCL EtO caused significant loss in molecular weight and number and changes to the surface morphology by SEM. EtO has also been shown in the literature to be an inappropriate sterilisation technique for biodegradable scaffolds due to changes in the structural and biochemical properties [17, 22]. For example, Hooper et al. showed that EtO of polycarbonate materials caused a reduction in yield strength and faster degradation rates compared to nonsterilised controls [25].

Although not considered sterilisation techniques for medical devices, we also evaluated the effect of different disinfectant methods. Techniques such as bleach, using antibiotics, and UV radiation are used in the laboratory setting to sterilise scaffolds for* in vitro* and in vivo examination. The use of SDIC prevented contamination of the samples and was not associated with any significant changes in mechanical properties or any visual deformation of both nanocomposite polymers. Although longstanding polymer exposure to bleach has been associated with increased hydrophobicity [26], we did not observe any significant changes in surface wettability of POSS-PCL scaffolds. Bleach has been shown to increase roughness and porosity of polymeric scaffolds [26]. In this study, we observed similar submicron changes to POSS-PCL scaffold structure, mainly an increase in the number of pits. SDIC was the sole sterilisation method, which showed slight changes in the surface chemistry of the scaffolds on the FTIR spectra, likely due to hydrolysis.

Ethanol and UV are all useful sterilisation techniques for laboratory analysis and provided sterility of the POSS-PCU and POSS-PCL nanocomposite scaffolds. For POSS-PCU scaffolds, UV and ethanol did cause some changes to the surface as shown by SEM although bulk mechanical properties were not affected. UV decreased the contact angle of POSS-PCU, which is consistent with other reports of UV creating hydrophilic surfaces [27–29]. UV sterilisation may have oxidized the surface and caused a drop in the water contact angle [27–29].

POSS-PCL scaffolds also maintained their bulk mechanical properties after ethanol and UV modification. However, reports in the literature show that whilst Gram-positive, Gram-negative, acid-fast bacteria, and lipophilic viruses show high susceptibility to concentrations of ethanol in water ranging from 60 to 80%, hydrophilic viruses and bacterial spores are resistant to the microbial effects of ethanol [30]. Antibiotic solution had minimal effect on the surface or bulk properties of POSS-PCU or POSS-PCL. Furthermore, reports have shown that different microorganisms have varying sensitivity to UV [19]. For example UV easily destroys vegetative bacteria but bacterial spores and prions are more resistant. Some viruses are considered inactivated whilst others are resistant [19]. Antibiotic solutions inactivate bacteria by interfering with DNA replication [19]. However, such solutions are only effective against vegetative bacteria and spores whilst fungi, moulds, and viruses are not affected [19]. Therefore, ethanol, UV, and antibiotics are considered to be chemical disinfectants instead of sterilising techniques and not used in the sterilisation of biomedical devices [19].

Plasma technology is defined as neutral ionised gas and includes photons, electrons, positive, and negative ions, atoms, free radicals, and nonexcited molecules [31]. Several types of plasma techniques exist but in this study radiofrequency plasma was utilised. Argon plasma demonstrated no changes in the material characteristics of both POSS-PCU including mechanical, molecular number and weight, surface chemistry by FTIR, or surface topography by SEM. However, argon plasma did cause some surface topographical changes of POSS-PCL due to potential etching effect of plasma treatment. The surface wettability significantly decreased after argon plasma for both POSS-PCU and POSS-PCL. The sterilisation process itself may affect the surface and bulk properties to negatively influence its cytocompatibility and biocompatibility. The* in vitro* compatibility of the sterilised scaffolds was performed to evaluate the release of cytotoxic molecular weight products from the sterilisation. Viability data demonstrated that significantly more cells were able to adhere to POSS-PCU and POSS-PCL after argon plasma surface modification compared to other sterilisation techniques by Day 7 (Figure 4). Several studies have found that decreasing the water contact angle of scaffolds below 90° due to plasma treatment induces a hydrophilic surface causing a greater number of cells adhere to the biomaterial surface [32, 33]. Both POSS-PCU and POSS-PCL became more hydrophilic after argon plasma surface modification, without causing bulk mechanical properties (mechanical and molecular number/weight). With optimal sterilisation efficacy, maintenance of mechanical properties, and improvement in cell biocompatibility, plasma demonstrated the optimal sterilisation process in this study.

Menashi et al. first reported the use of argon for sterilisation in 1968 after sterilising the surface of vials contaminated by bacterial spores [34]. Since then the gas type and the power of discharge have shown to influence the efficacy of the treatment. Analysis of RF plasmas has suggested that the chemical species created in the discharge are responsible for the destruction of the microorganisms and the UV or thermal effects are secondary effects [35, 36]. Argon plasma has shown to sterilise titanium implant surfaces [37]. Other types of plasma have been investigated for the sterilisation of materials. The comparison of the ethanol, dry oven, autoclave, UV radiation, and hydrogen peroxide plasma as sterilisation techniques for electrospun PLA scaffolds [38] showed that UV irradiation and hydrogen peroxide were the most effective without affecting the chemical and morphological features. The authors demonstrated the hydrogen peroxide produced destructive hydroxyl free radicals, which can attack membrane lipids, DNA, and other essential cell components to deactivate the microorganisms [38].

There were limitations to this work. During the plasma treatment the scaffolds were exposed to the unsterile environment whilst moving them from the plasma chamber. To further optimise the plasma treatment the apparatus will be placed in a sterile laminar flow cabinet in the future.

In summary, argon plasma maintained the properties of the nanocomposite scaffolds in addition to improving cell adhesion (Figure 4). Plasma sterilisation is easily transferrable to clinical practice for sterilisation of materials as it is simple to operate and the lack of toxic residuals makes it safe for the operator.

## 5. Conclusion

In conclusion, the FDA approved sterilisation technique of autoclaving maintained POSS-PCU characteristics but was unsuitable for biodegradable POSS-PCL scaffolds. Taking all results into account, we argue that plasma surface modification using argon gas may effectively sterilise polyurethane biomaterials as well as improve cytocompatibility of tissue engineering scaffolds by modification in surface wettability and topography. Argon plasma should be explored and optimised for the sterilisation of further biomaterials.

## Figures and Tables

**Table 1:** **Mechanical Properties of POSS-PCU and POSS-PCL after sterilisation processes.** Young's modulus, maximum stress, elongation at break, and thickness of control POSS-PCL and POSS-PCU and samples subjected to ethanol, bleach (SDIC), plasma, ethylene oxide, UV radiation, antibiotic/antimycotic treatment, and gamma irradiation.

**Sterilisation Method**		**Control**	**Bleach (SDIC)**	**Ethanol**	**Gamma**	**Ethylene Oxide**	**UV**	**Antibiotic/Antimycotic**	**Plasma**	**Autoclaving**
Young's Modulus (MPa)	POSS-PCL	0.18 ± 0.0	0.29 ± 0.02	0.20 ± 0.02	0.32 ± 0.04	0.40 ± 0.11	0.35 ± 0.02	0.25 ± 0.03	0.30 ± 0.05	N/A
	POSS-PCU	0.55 ± 0.04	0.57 ± 0.02	0.56 ± 0.02	0.56 ± 0.04	0.55 ± 0.02	0.55 ± 0.02	0.53 ± 0.01	0.55 ± 0.03	0.54 ± 0.02
Maximum Stress (MPa)	POSS-PCL	0.56 ± 0.08	0.61 ± 0.10	0.31 ± 0.11	1.17 ± 0.15	1.71 ± 0.19	1.45 ± 0.04	1.10 ± 0.14	0.80 ± 0.26	N/A
	POSS-PCU	0.83 ± 0.03	0.82 ± 0.06	0.85 ± 0.03	0.83 ± 0.01	0.84 ± 0.01	0.83 ± 0.01	0.81 ± 0.12	0.83 ± 0.02	0.82 ± 0.04
Elongation at break (%)	POSS-PCL	589.6 ± 26.2	549.7 ± 68	293.0 ± 61	462.2 ± 16	471.6 ± 39	387.5 ± 22	469.5 ± 11	500.1 ± 30	N/A
	POSS-PCU	283.3 ± 9.57	273.6 ± 10.01	279.2 ± 1.74	288.0 ± 3.05	288.6 ± 1.46	269.3 ± 8.55	273.8 ± 9.01	281.1 ± 4.12	280.5 ± 7.88
Thickness (mm)	POSS-PCL	2.0 ± 0	1.43 ± 0.15	1.525 ± 0.13	1.6 ± 0.22	1.2 ± 0.25	1.38 ± 0.05	1.55 ± 0.17	2.433 ± 0.12	N/A
	POSS-PCU	0.75 ± 0.04	0.77 ± 0.02	0.84 ± 0.02	0.77 ± 0.05	0.76 ± 0.02	0.77 ± 0.02	0.75 ± 0.03	0.76 ± 0.02	0.74 ± 0.06

**Table 2:** **Summary of gel permeation chromatography (GPC) results**. Molecular weight (*M*_w_) and molecular number (*M*_n_) values of POSS-PCL and POSS-PCU after different sterilisation techniques.

**Sterilisation Method**		**Control**	**Bleach (SDIC)**	**Ethanol**	**Ethylene Oxide**	**Gamma**	**Plasma**	**Antibiotic/Antimycotic**	**Autoclaving**	**UV**
Mass-average Molecular Weight (Mw) (g/mol)	POSS-PCL	361100	350900	356100	276300	151200	358600	391600	174600	365400
	POSS-PCU	91200	92000	89400	112200	135000	90800	92700	88300	93800
Number-average Molecular Weight (Mn) (g/mol)	POSS-PCL	141000	137600	111300	97500	44700	113300	160800	88100	155300
	POSS-PCU	47700	46300	47200	34200	40000	46700	46200	45500	46200

**Table 3:** **Summary of POSS-PCL and POSS-PCU sterilisation efficacy for bleach (SDIC), ethanol, ethylene oxide, gamma, plasma, antibiotic/antimycotic, UV, and autoclaving sterilisation techniques.** All control samples (3) of POSS-PCU and POSS-PCL and 1 of 9 POSS-PCL samples sterilised using UV and antibiotic/antimycotic were infected.

**Sterilisation Method**		**Control**	**Bleach (SDIC)**	**Ethanol**	**Ethylene Oxide**	**Gamma**	**Plasma**	**Antibiotic/Antimycotic**	**UV**	**Autoclaving**
POSS-PCL	TSB	9/9	0/9	0/9	0/9	0/9	0/9	1/9	1/9	N/A
	THY	9/9	0/9	0/9	0/9	0/9	0/9	1/9	1/9	N/A
POSS-PCU	TSB	9/9	0/9	0/9	0/9	0/9	0/9	0/9	0/9	0/9
	THY	9/9	0/9	0/9	0/9	0/9	0/9	0/9	0/9	0/9

## Data Availability

The data used to support the findings of this study are available from the corresponding author upon request.
